# Increasing ductility beyond post-uniform deformation through Zn lamellae deformation in Al at room temperature

**DOI:** 10.1016/j.heliyon.2024.e34984

**Published:** 2024-07-20

**Authors:** Seung Zeon Han, Byungki Ryu, Il-Seok Jeong, Sung Hwan Lim, Eun-Ae Choi

**Affiliations:** a Extreme Materials Institute, Korea Institute of Materials Science (KIMS), Changwon, 642-831, South Korea; b Energy Conversion ResearchCenter, Korea Electrotechnology Research Institute (KERI), Changwon, 51543, South Korea; c Department of Advanced Materials Science & Engineering, Kangwon National University, Chuncheon, 200-701, South Korea

**Keywords:** Al-Zn alloy, Interface, Discontinuous precipitation, Zn lamellae, Deformation

## Abstract

The Zn element precipitates during aging in the Al–Zn binary alloy. Increased Zn content and prolonged aging leads to discontinuous Zn precipitation. The addition of 2 wt% Cu to the Al-43 wt%Zn alloy accelerates this discontinuous precipitation, resulting in decreased thickness of Zn layers and inter-distance between them. This acceleration is attributed to the influence of Cu solutes on the Zn phase, thereby reducing the interface energy between Zn precipitates and the Al matrix. The Al–Zn–Cu alloy demonstrates exceptional behavior during tensile tests, displaying a simultaneous increase in tensile strength and ductility alongside an 75 % reduction in area at room temperature drawing. Notably, despite the drawn beyond uniform deformation limit, there is an observed increase in total elongation. Our demonstration highlights this phenomenon, attributing it to the sustained coherent interface between the Zn layer and the Al matrix, as well as the uninterrupted continuity of Zn layers during drawing.

## Highlights

• The discontinuously precipitated Zn phase plastically elongated without breaking in Al matrix.
• The semi-coherent interface relationship between Zn and Al phase was maintained even after cold drawn.
• The Al composite with lamellar Zn phases not only increase strength and total ductility after cold drawn.

## Graphical abstract

**Figure:**
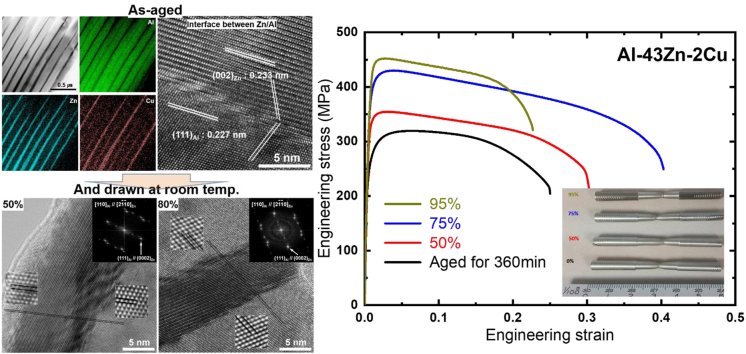

## 1 Introduction

The metallurgists have long been seeking for metals with high strength and ductility, since structural metals require high strength for safety and high ductility for easy fabrication [1]. There are a number of ways to increase the strength of metal, including work hardening [2], grain refinig [3] and particle hardening [4]. Unfortunately, all of these conventional methods of increasing strength either greatly sacrifice ductility or do so to a lesser extent. Numerous attempts have been made to achieve this goal through the manipulation of metal microstructures, including the implementation of ultra-fine grain structures [[5], [6], [7], [8], [9]], nano-twin structures [[10], [11], [12], [13]], marble structures [14,15], and bimodal intermetallic compounds [[16], [17], [18], [19]].

Among the various alloys strengthened by hardening mechanisms, precipitation hardening alloys, notably aluminum alloys [[20], [21], [22]], are commercially recognized for their ease of fabrication [1,23]. Nevertheless, cold working processes after aging, such as drawing or rolling unavoidably result in a decrease in ductility, despite enhancing the overall strength. Meanwhile, the Al–Zn binary alloy exemplifies a typical precipitation-type alloy, wherein zinc precipitates at lower temperatures, after establishing a single-phase region that forms a solid solution at higher temperatures [23]. To increase strength, increasing the volume fraction of the Zn phase to be precipitated is an easy approach. However, higher amounts of added zinc result in an increased precipitation driving force, which leads to discontinuous zinc precipitation during aging and consequently causes a rapid decline in strength [23].

While investigating the aging and subsequent deformation characteristics of Al–Zn–Cu alloys, our findings revealed that specimens work-hardened at room temperature exhibited greater strength and ductility compared to their counterparts in the as-aged state. This is extremely unique phenomenon since ductility drop is inevitably accompanied with cold working. It is more exciting that we can obtain high strength and ductility combination in commercially available Al–Zn–Cu alloys simply by not-so-novel techniques of casting, aging and drawing. For this, we deliberately utilized the non-conventional microstructure of discontinuous precipitate, which later turned into lamellar precipitate, by over-aging. To elucidate the concurrent enhancement of strength and ductility resulting from cold working, our investigation focused to the influence of additive composition—particularly Cu—in the Al–Zn binary alloy. Our study centers on understanding the morphological alterations of precipitates following aging and subsequent cold working and their effect on the tensile strength and ductility, employing simple first-principle calculations.

## 2 Experimental and calculation procedure

Pure Al and Zn with purities of 99.9 % and Cu with a purity of 99.99 % were used to prepare the alloy. The nominal compositions of the alloys were designed to induce normal and discontinuous precipitation, and they are shown in Table 1.

**Table 1:** Average composition of aluminum alloys used in this work (wt.%).

Alloys	Al	Zn	Cu
Al–45Zn	Bal.	43.4	–
Al–43Zn–2Cu	Bal.	42.4	2.55

The Al–Zn alloy with and without additional Cu were fabricated to 25 mm-thick cast ingots, via electric resistance furnace melting. And they were homogenized at 400 °C for 24 h, and then cylindrically machined with a diameter of 20 mm and a length of 200 mm. To get the sufficient length for drawing process, all specimens were swaged with a 75 % reduction of cross-sectional area with intermediate annealing at 400 °C for 15 min at 20 % of their reduction during swaging. To eliminate the thermomechanical history of the specimens, both Al–Zn alloys without or with Cu were subsequently solution heat-treated at 400 °C for 60 min and then water quenched, and followed by aging at 160 °C. Finally, the two types of specimens, in which the precipitates were uniformly distributed and fully precipitated discontinuously, were aged for 15 and 360 min respectively. To align the plate-like precipitates in a single direction, a drawing process was applied to alloys that were aged under the same conditions of 160 °C for 360 min after being solutionized. Cylindrically machined specimens with diameters of 5 mm, were drawn at room temperature with a 50, 75, 80 and 95 % reduction in their cross-sectional area (true strain, ɳ = 0.7, 1.4, and 3.0).

Tensile tests were performed with a gage length of 10 mm at a nominal strain rate of 2 × 10^−1^/s on a universal testing machine (4206, Instron, USA). The grain morphologies and secondary-phase precipitates of the aged specimens were observed with scanning electron microscope (JSM-6610LV, JEOL, Japan). The precipitates were investigated using a 200-kV field-emission transmission electron microscope (JEOL-2100F, JEOL, Japan) equipped with an energy-dispersive X-ray spectroscopy (EDS) detector, and a scanning transmission electron microscope. The TEM specimens were prepared to have a 3-mm diameter in the form of 60-⌠m-thick disk-type plates via mechanical polishing with a digitally enhanced precision specimen grinder (DEPS-101, Total Solution) and then ion-polished with a precision Ar ion polishing system (691 PIPS, Gatan, USA). The parallel and perpendicular sections for confirmation of the lamellar nature of discontinuous Zn precipitates were conducted with a dual beam-focused ion beam (FIB, Helios nanolab, Netherlands).

Density Functional Theory (DFT)-based first-principles were conducted to investigate the effect of Cu addition on the elastic properties and crystal structure of Zn precipitates. Special Quasi-Random Structures (SQS) [[24], [25], [26]] were used to create a hexagonal close-packed (HCP) (4 × 4 × 2) Zn supercell consisting of 64 atoms with Cu concentration up to 11 wt%. In this model, Cu atoms stochastically replaced Zn atoms to ensure randomness up to the fourth-nearest neighbors, with the correlation function below 0.05.

The Projector Augmented-Wave (PAW) method [27,28], incorporating the 3d electrons of Zn and Cu, was employed with a 550 eV cut-off energy. Our computational approach utilized the Perdew-Burke-Ernzerhof (PBE) approximations [29] for the exchange-correlation potential, as implemented within the Vienna ab initio simulation package (VASP) [30,31]. Gamma meshes of 7 × 7 × 7 for structure optimization and 9 × 9 × 9 for elastic property calculations were employed, respectively, and a force-convergence criterion of 0.01 eV/Å.

The elastic properties of the supercell were calculated using the ELASTOOL program [32]. Due to the Cu-induced disruption of symmetry, a triclinic system was adopted. Strains from −0.06 to 0.06 with intervals of 0.03 were applied to derive the stiffness tensor from the stress-strain relationship [33], enabling the determination of the polycrystalline elastic properties.

## 3 Results and discussions

In many precipitation-hardening alloys, two distinct types of precipitates commonly form: continuously precipitated particles and discontinuously precipitated ones [[34], [35], [36], [37], [38], [39], [40], [41]].

Al–Zn binary alloy also has two type of precipitation process, that is, continuous precipitation (CP) and discontinuous precipitation (DP), and the formation of these precipitates—continuously and discontinuously precipitated particles—is influenced by the alloy's composition and the specific temperature and time conditions during thermomechanical treatment. DP [34] is favored, especially when a substantial quantity of components contributing to the precipitated phase is added [23].

**Fig. 1:**
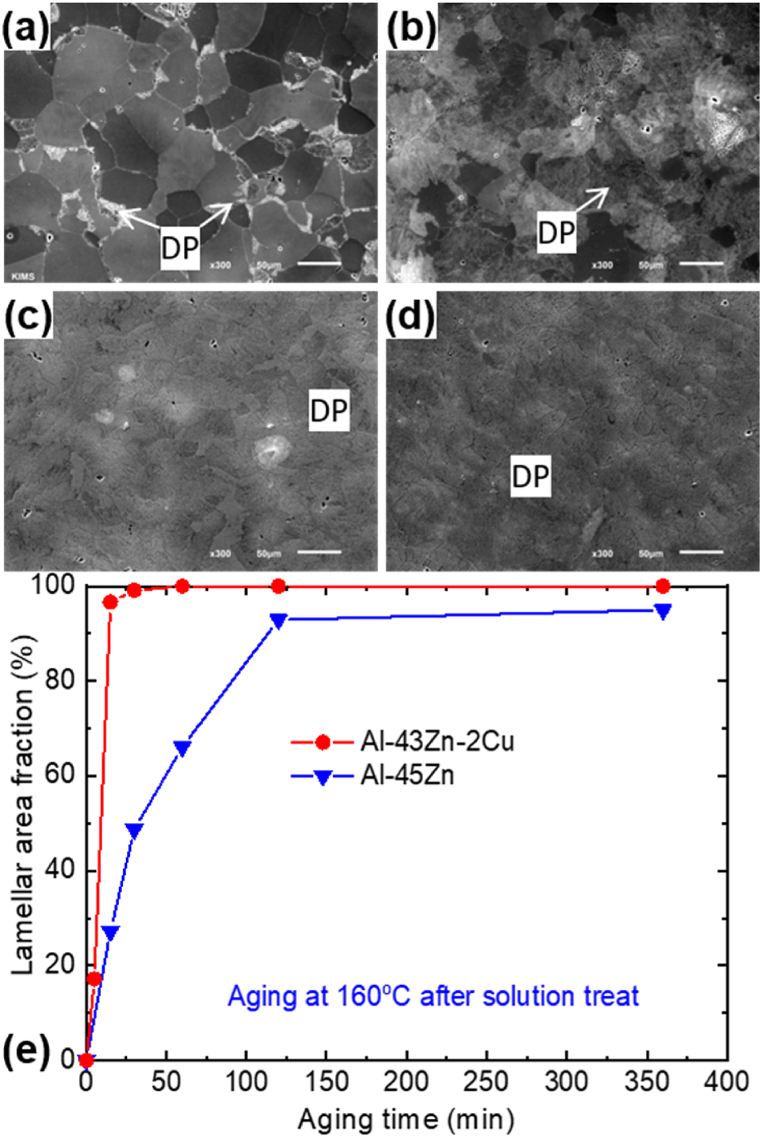
Microstructure comparison of Al–45Zn and Al–45Zn–2Cu alloys after solution treatment and subsequent aging at 160 °C for (a, c) 15 min and (b, d) 360 min. (e) Variation in the area fraction of discontinuously precipitated regions with increasing aging time.

**Fig. 2:**
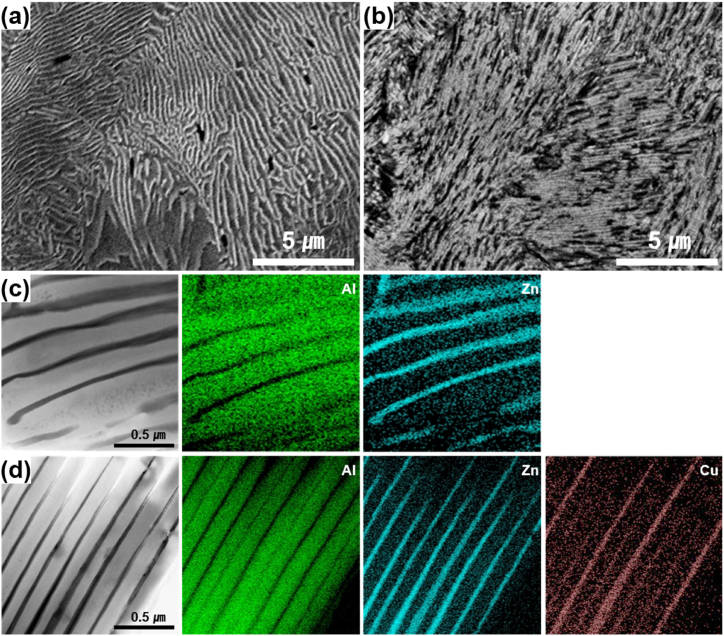
The microstructure of discontinuous precipitates in (a) Al–45Zn and (b) Al–43Zn–2Cu alloy is shown in Fig. 1(c) and (d). Low-magnification TEM images are provided for (c) Al–45Zn and (d) Al–43Zn–2Cu alloy, respectively.

After undergoing solution heat treatment and aging, the Al-45 wt% Zn alloy, containing a substantial amount of Zn, exhibited DP, as shown in Fig. 1. The substitution of 2 wt% Cu for Zn demonstrated an accelerated DP, leading to the complete development of DP regions after aging for 360 min in that alloy. The addition of 2 wt % Cu in the Al–43Zn alloy potentially facilitates the precipitation of AlCuZn intermetallic compounds. These compounds may act as inoculants for precipitates, particularly during the over-aging process. Furthermore, as shown in Fig. 2 (the magnified image of Fig. 1(c) and (d)), following a 360-min aging, the width of the Zn phases formed through DP and the inter-distance between these Zn phases within the Al matrix decreased concurrently due to the addition of Cu to the Al–Zn alloy.

**Fig. 3:**
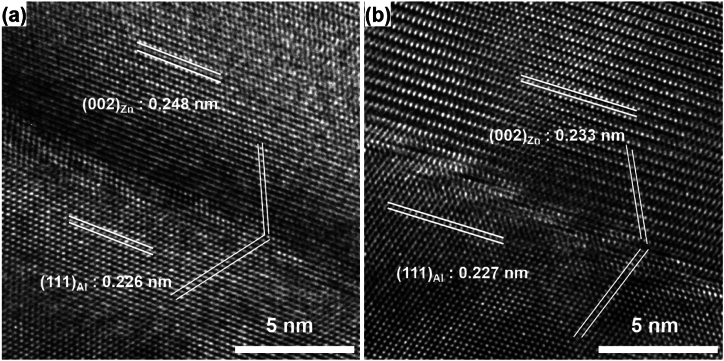
High-magnification TEM images for (a) Al–45Zn and (b) Al–43Zn–2Cu alloy showing the lattice spacing of Al and Zn phase.

**Fig. 4:**
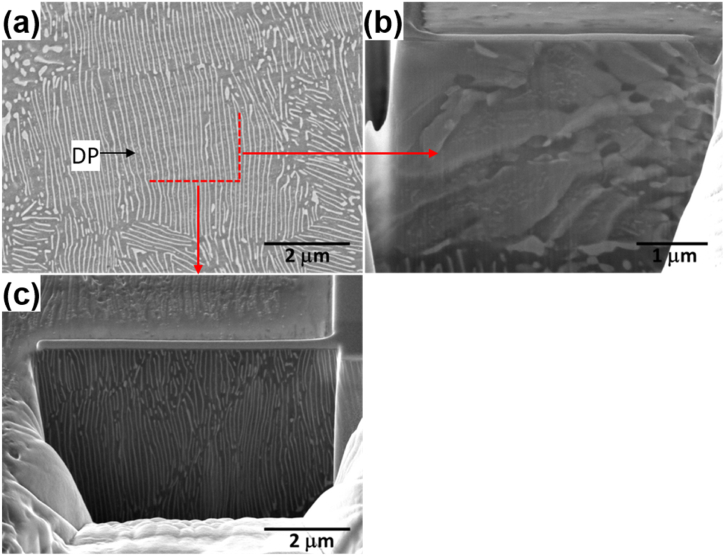
(a) Vertical section of Al–Zn–Cu with red dotted lines denoting the FIB cutting direction. FIB-sectioned region shown in (b) parallel and (c) vertical views, displaying the lamellar Zn precipitates.

The high-magnification image describing the interface between Zn formed by DP and the Al matrix reveals a semi-coherent relationship with the (0002)_Zn_/(111)_Al_ planes, as shown in Fig. 3. This configuration results in the retention of a chemically strong atomic bond at the interface. The SEM observation, employing the FIB technique, substantiated the lamellar structure of Zn formed by DP in the Al–Zn–Cu alloy, as shown in Fig. 4. This is attributed to the stability of the interface between the Zn precipitate and the Al matrix, characterized by the (0002)_Zn_/(111)_Al_ relation, which is more stable than any other interface relation between Zn and Al. Consequently, the growth of lamellar Zn facilitated a reduction in total interface energy during the aging. Especially, the added copper was dissolved in the Zn precipitated phase, and it further decreased the interface energy of the interface between (0002)_Zn_ and (111)_Al_. Conclusively, the thickness of Zn lamellae and the inter-distance between them are decreased.

**Fig. 5:**
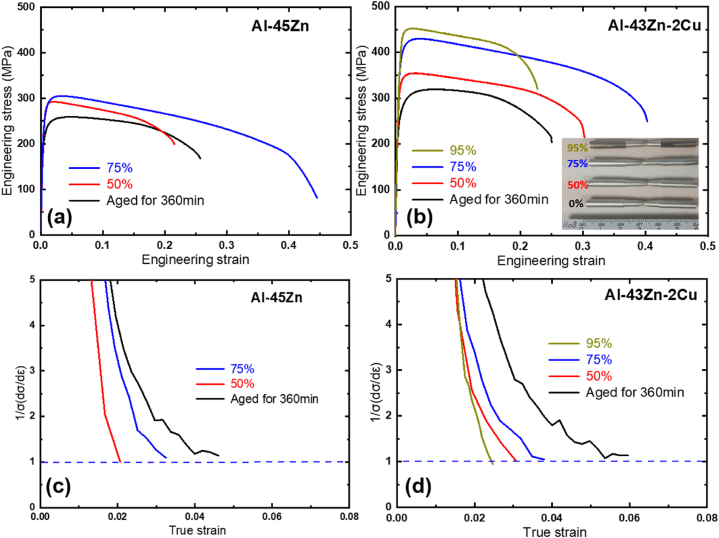
Engineering stress-strain curves of aged and cold-drawn (a) Al–45Zn and (b) Al–43Zn–2Cu alloys, along with true strain rate normalized by true strength for (c) Al–45Zn and (d) Al–43Zn–2Cu alloys.

Room temperature drawing was applied to fully aged Al–Zn specimens, both with and without Cu, entirely covered by DP as shown in Fig. 2. The stress-strain curves for these specimens are shown in Fig. 5. To mitigate any scale effects during the tensile test, specimens of identical sizes as shown in the photo within Fig. 5B were used. The alloy fully covered by DP exhibits not only increased strength but also enhanced ductility following a 75 % area reduction during drawing, reaching a true strain of 1.38. Specifically, the Al–Zn alloy containing Cu demonstrated a gradual simultaneous increase in both strength and ductility. However, subsequent further drawing with a 95 % area reduction (true strain: 2.3) resulted in increased strength but a subsequent decrease in ductility. Fig. 5 (c) and (d) display the strain rate normalized by strength. A y-axis value of 1 represents the limit of uniform deformation, highlighting a decrease in actual ductility resulting from uniform deformation, despite an observed increase in total elongation during cold-drawing.

Contrary to the typical behavior observed in cold-worked metals, where strength rapidly decreases after the necking point, the gradual decrease in strength alongside an increase in total elongation holds significant meaning. This is because the area under the stress-strain curve is a measure of toughness, representing the energy absorbed by a material during deformation.

**Fig. 6:**
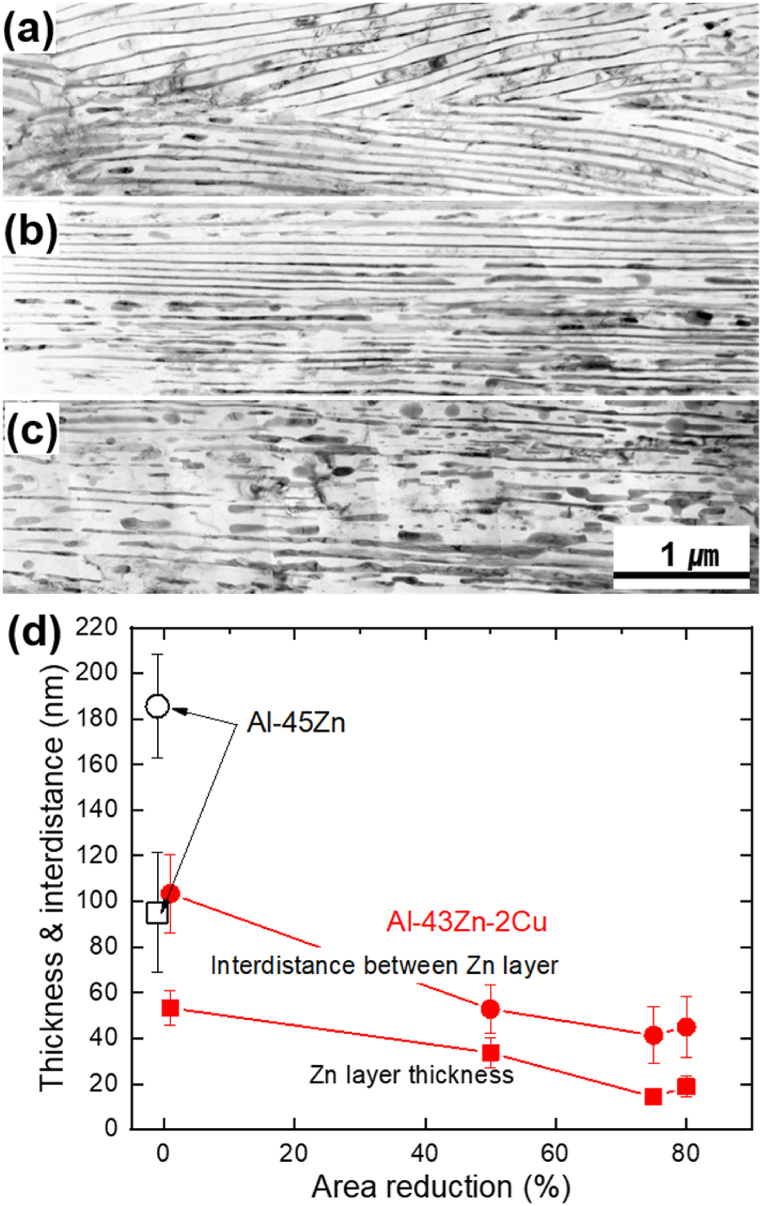
Zn layer structure observed in aged and successively drawn Al–Zn–Cu alloy at reduction ratios of (a) 50 %, (b) 75 %, and (c) 80 %. (d) Variation in the thickness of Zn layers and the interdistance between them with increasing drawing ratios at room temperature.

**Fig. 7:**
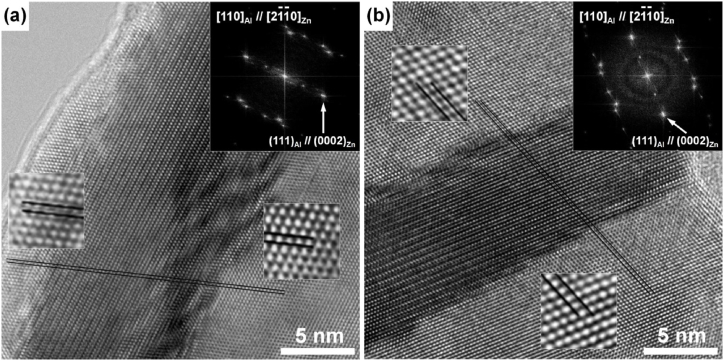
The detailed HRTEM image and a Fast Fourier transform (FFT) diffractogram of plastically deformedAl-Zn-Cu alloy with drawing of (a) 50 % and (b) 80 % of area reduction.

To understand this unique phenomenon where the Al alloy with a fully DP structure exhibits concurrent increases in strength and total elongation despite a decreased uniform deformation region, TEM analysis was conducted, and the findings are presented in Fig. 6. As the drawing ratio increased, the Zn layer formed by DP exhibited alignment along the drawing direction, accompanied by a decrease in both the thickness of the Zn layer and the inter-distance between them, as illustrated in Fig. 6. Notably, at approximately 80 % area reduction, the continuity of the Zn layer was disrupted, resulting in the formation of spherical Zn particles. This result implies that the increase in strength correlates with the reduction in both the thickness of the Zn layer and the distance between them. Moreover, the observed increase in ductility beyond the uniform deformation limit could be attributed to the preservation of the continuity of the Zn layer during the drawing process. The occurrence of spheroidization instead of the anticipated breaking of the Zn layer around the 80 % area reduction may seem unexpected. Considering the melting points of Al and Zn at 660 °C and 419 °C, respectively, the room temperature corresponds to approximately 32 % and 43 % of the homologous temperature of Al and Zn, respectively. Furthermore, the heat generated during drawing could contribute to disrupting the continuity of the Zn layer, aiding in the formation of spherical Zn particles. The findings presented in Fig. 6 demonstrate the plastic deformation of the Zn layer formed through DP during the drawing process of the Al–Zn–Cu alloy. However, as illustrated by Fig. 7, the coherent relationship between the Zn layer and the Al matrix remains even after an 80 % area reduction resulting from drawing.

Upon increased cold-working, the initially nano-sized Zn lamellae within the Al matrix further elongated and aligned unidirectionally along the drawing axis, demonstrated in Fig. 6. However, extensive cold-working over the 80 % led to the fragmentation of Zn lamellae bundles, resulting in the formation of spheroidized Zn particles, shown in Fig. 6 (c). This change correlates with the onset of decreasing total ductility observed at 95 % cold-working, as depicted in Fig. 5(b). This highlights the significance of maintaining the continuity of Zn lamellae for enhancing ductility through cold-working.

The addition of Cu notably reduces interface energy, consequently decreasing inter-distance between the Al matrix and Zn lamellae. This reduction of inter-distance limits the available space for deformation within the material. During tensile elongation, stress tends to concentrate at the interface between the Al matrix and the Zn layer. The increased interface area due to reduced spacing provides multiple sites for stress concentration. As a result, stress distributes more evenly, even beyond the limit of uniform deformation during tensile testing. Ultimately, the reduced lamellar spacing significantly contributes to creating numerous sites of localized deformation within the material, particularly within the Al matrix.

Again, the smaller lamellar spacing is the reason why localized deformation does not occur significantly within the material. We therefore conclude that this robust coherency between the relatively hard Al matrix and the soft Zn lamellae significantly contributes to sustaining total ductility, despite the decreased uniform deformation in the cold-worked Al–Zn–Cu alloy. In contrast to composite materials, coherent Zn lamellae generate a significant amount of coherent strain energy at the interface. This strain energy field impedes dislocation movement within the Al matrix, providing additional strength to the alloy. When the alloy rod increases four-fold while maintaining a constant volume, the increase in simple calculation suggests a four-fold increase in the interfacial area between the Al matrix and Zn lamella with 75 % cold-working, equating to a true strain of 1.386 (η). The increased coherent interface between Zn lamellae and Al matrix facilitates the ductility of cold-worked Al–Zn–Cu alloy by absorbing considerable strain during tensile loading until the breakage of Zn lamellae. Subsequently, the sustained coherence between the Al matrix and Zn lamellae suppresses localized plastic deformation within the Al matrix. The enhanced post-necking, resulting from the suppressed local deformation, contributes to the overall ductility increase upon cold-working. From the viewpoint of strengthening, the well-known particle strengthening theory states that the inter-distance between particles greatly influences the strengthening. Therefore, a smaller inter-distance between particles leads to higher strength in particle-hardened alloys [1,4,23]. Our previous work revealed that strengthening occurred when the inter-distance between uniaxial, discontinuously precipitated fibrous precipitates was decreased [42,43]. As shown in Fig. 6, the decrease in inter-distance between Zn lamellae has a similar effect to the decrease in inter-distance between particles, resulting in increased strengthening.

While HCP (Hexagonal Closed Packed) Zn, the precipitates found in Al–Zn alloy, are commonly understood to be softer than FCC (Face Centered Cubic) Al, the Al–45%Zn alloy demonstrates a notable increase in strength [23]. According to the particle strengthening theory, deformable particles are expected to provide less effective strengthening due to the ease with which dislocations can pass through them. Understanding the mechanism how softer Zn precipitates in an Al matrix contribute to increased strength is necessary. Investigating why these soft particles, like Zn, enhance the strength of aluminum despite their inherent softness is crucial.

**Fig. 8:**
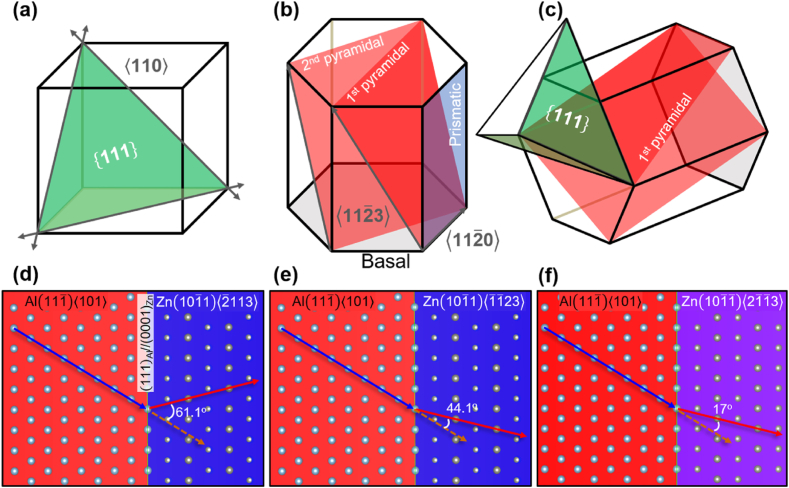
Crytal structure of (a) FCC Al, (b) HCP Zn and (c) their mathing plain having coherent relation. (d), (e) and (f) basal slip direction of Al and slip direcion on the Zn's various 1st pyramidal plane.

Fig. 8 illustrates the crystal structures of Al and Zn, demonstrating a coherent relationship established by previous research [23]. Along the interface of (111)_Al_ and (0002)_Zn_, each unit cell is depicted to match, as detailed in Fig. 8 (c). Furthermore, Fig. 8(d–f) illustrate the respective slip directions of Al and Zn across this coherent interface. The distinct orientations of the basal slip planes for zinc (Zn) and aluminum (Al), as depicted in Fig. 8 (c), suggest a significant mismatch that could hinder or make improbable the slip from the basal plane of Al to the basal plane of Zn. This discrepancy in the orientations of their basal planes may impede any form of slip or deformation directly from the basal plane of Al to that of Zn due to the mismatch in their crystallographic structures.

**Fig. 9:**
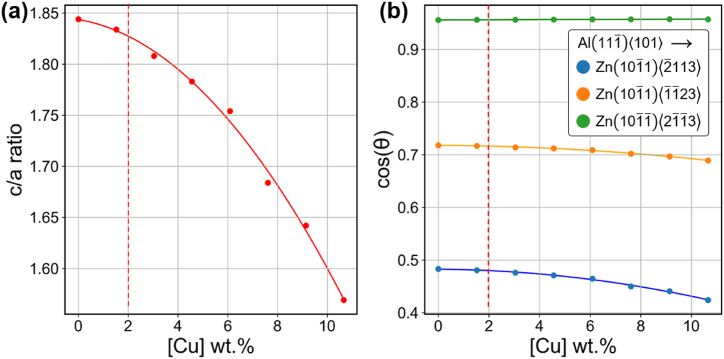
Variation of (a) c/a ratio and (b) cosine of slip directions between FCC Al and HCP Zn with increasing Cu addition. The dashed vertical red line indicates the 2 wt% Cu addition utilized in this study.

Consequently, the potential for slip between the basal plane of Al and the second slip plane of Zn emerges, as shown in Fig. 8(d)–(f). However, due to the substantial Burgers vector of the second slip plane and the considerable angle required for the slip transition between Al and Zn, the possibility arises for dislocation pile up at their interface. This accumulation, despite the presence of a soft-phase precipitate, could lead to a notable enhancement in strength within the Al–Zn interface. As previously discussed, the introduction of copper into the Al–Zn alloy serves to reduce the interface energy between (111)_Al_ and (0002)_Zn_ [23]. This reduction in interface energy leads to a decreased inter-distance between Zn lamellae, consequently enhancing both the strength and ductility of the alloy. "By the way, the question arises regarding whether the dissolved copper element in zinc solely serves to decrease the interfacial energy between Zn and Al. To address this question, the discussion focuses on the influence of copper solutes within the zinc phase on the c/a ratio of zinc and the angle of slip direction between each phase with increasing Cu addition, as illustrated in Fig. 9.

The c/a values derived from DFT calculations demonstrate a consistent decrease with increasing Cu content, as depicted in Fig. 9(a). Conversely, there was no significant alteration of the cos(θ) value relative to the amount of Cu added in this study. The cos(θ) serves as an indicator of the force that induces slip in the aluminum phase and its reduction when slip is transferred to the Zn phase, illustrated in Fig. 8(d)–8(f). The alloy with 2 % Cu addition, as indicated by the DFT calculation results in Fig. 9, exhibited negligible changes in c/a and cos(θ) values compared with Al–Zn alloy without Cu. Additionally, the bulk modulus, shear modulus, Young's modulus, and Poisson's ratio of the Al–Zn alloy, both without and with Cu, were calculated, and the results are presented in Table 2. The addition of Cu to the Al–Zn alloy in this study did not significantly change its elastic properties. Consequently, it can be concluded that the atomic structure undergoes minimal change, particularly regarding the interface energy between Zn precipitates and the Al matrix [23]. This supports the conclusion that the primary factor influencing the strength and ductility of the Al–Zn alloy is associated with the interface energy between Zn precipitates and the Al matrix.

**Table 2:** Elastic properties of HCP Zn with Cu addition calculated by DFT. Bulk (B), Shear (G), Young's (E) modulus and Poisson's ratio (ν) represented, respectively.

Cu (wt.%)	B (GPa)	G (GPa)	E (GPa)	ν
0.00	81.2	40.1	103.3	0.288
1.52	81.0	39.3	101.5	0.291

## 4 Summary

The addition of Cu to the Al–Zn alloy reduces the interface energy between the discontinuously precipitated Zn lamellae and the Al matrix. Consequently, it leads to a simultaneous increase in both strength and total elongation to failure with an increasing drawing ratio during room temperature drawing. The added Cu primarily exists as a solute in the Zn precipitated phase rather than within the Al matrix. While it decreases the interface energy, it does not significantly alter the crystal structure or inherent properties, such as shear or bulk modulus. The primary factor contributing to the simultaneous increase in strength and total elongation to failure is attributed to the reduced thickness of Zn lamellae and the decreased inter-distance between them. However, as the drawing ratio surpasses 80 % of area reduction, the continuity of the Zn lamellae is broken, leading to an increase in strength accompanied by a decrease in ductility.

## CRediT authorship contribution statement

**Seung Zeon Han:** Writing – review & editing, Writing – original draft, Validation, Supervision, Project administration, Investigation, Funding acquisition, Conceptualization. **Byungki Ryu:** Validation, Software, Investigation, Data curation. **Il-Seok Jeong:** Visualization, Software, Methodology. **Sung Hwan Lim:** Visualization, Investigation, Data curation. **Eun-Ae Choi:** Writing – review & editing, Writing – original draft, Visualization, Validation, Software, Formal analysis, Conceptualization.

## Declaration of competing interest

The authors declare no competing interests.
