# Supplementary material for: Increasing ductility beyond post-uniform deformation through Zn lamellae deformation in Al at room temperature
Source: Heliyon. 2024 Jul 20;10(14):e34984. doi: 10.1016/j.heliyon.2024.e34984 (PMC11325366; doi:10.1016/j.heliyon.2024.e34984)
Supplement: Multimedia component 1 [file mmc1.docx]

**Prime novelty statements**

We observed a simultaneous increase in both the strength and ductility of over-aged Al-Zn alloys with discontinuous Zn lamellae upon cold-working at room temperature, in contrast to the conventional understanding that cold working typically renders metals brittle. The reason for the concurrent increase in strength and total ductility, even after room temperature working, despite a decrease in the uniform deformation limit compared to the aged counterpart, can be elucidated as follows. The coherence between the Zn lamellar precipitate and the Al matrix persisted even after cold rolling. The uninterrupted continuity of Zn lamellae during cold working directly correlates with the simultaneous increase in strength and ductility. The presence of Cu solute in Zn lamellae reduces interfacial energy, leading to a decrease in the inter-distance between lamellae and enhancing strength. Furthermore, it diminishes the space where stress concentration occurs, thereby suppressing necking, even when deformation extends far beyond the uniform deformation limit. Based on the results of Density Functional Theory (DFT) calculations, it was observed that the presence of Zn solute in the Zn phase did not alter the crystal structure or inherent properties such as bulk modulus. Consequently, it is concluded that the interface energy between Zn and Al predominantly influences the strength and overall ductility.
